# A Comprehensive Expression Profile of MicroRNAs in Porcine Pituitary

**DOI:** 10.1371/journal.pone.0024883

**Published:** 2011-09-28

**Authors:** Hongyi Li, Qianyun Xi, Yuanyan Xiong, Xiao Cheng, Qien Qi, Lin Yang, Gang Shu, Songbo Wang, Lina Wang, Ping Gao, Xiaotong Zhu, Qingyan Jiang, Yongliang Zhang, Li Yuan

**Affiliations:** 1 College of Animal Science, South China Agricultural University, Guangzhou, China; 2 School of Life Sciences, Key Laboratory of the Ministry of Education for Cell Biology and Tumor Cell Engineering, Xiamen University, Xiamen, China; 3 College of Life Sciences, Sun Yat-sen University, Guangzhou, China; University of Georgia, United States of America

## Abstract

MicroRNAs (miRNAs) are an abundant class of small RNAs that regulate expressions of most genes. miRNAs play important roles in the pituitary, the “master” endocrine organ.However, we still don't know which role miRNAs play in the development of pituitary tissue or how much they contribute to the pituitary function. By applying a combination of microarray analysis and Solexa sequencing, we detected a total of 450 miRNAs in the porcine pituitary. Verification with RT-PCR showed a high degree of confidence for the obtained data. According to the current miRBase release17.0, the detected miRNAs included 169 known porcine miRNAs, 163 conserved miRNAs not yet identified in the pig, and 12 potentially new miRNAs not yet identified in any species, three of which were revealed using Northern blot. The pituitary might contain about 80.17% miRNA types belonging to the animal. Analysis of 10 highly expressed miRNAs with the Kyoto Encyclopedia of Genes and Genomes (KEGG) indicated that the enriched miRNAs were involved not only in the development of the organ but also in a variety of inter-cell and inner cell processes or pathways that are involved in the function of the organ.

We have revealed the existence of a large number of porcine miRNAs as well as some potentially new miRNAs and established for the first time a comprehensive miRNA expression profile of the pituitary. The pituitary gland contains unexpectedly many miRNA types and miRNA actions are involved in important processes for both the development and function of the organ.

## Introduction

MicroRNAs (miRNAs) are a family of small RNAs that function as regulators of messenger RNAs [1]. Their discovery has revealed a new level of gene regulation in eukaryotes [2]. In animals, miRNAs regulate gene expression mainly by sequence-specific targeting of the 3′untranslated regions of target mRNAs, which usually results in repression of the gene expression [3]. Occasionally, miRNAs can suppress or activate genes by causing histone modification and DNA methylation of promoter sites [4], [5], or by targeting gene promoters [6]. miRNAs may target about 60% of mammalian genes [7] and have been shown to be involved in a wide range of biological processes including development, differentiation, proliferation, and immune response [8], [9], [10], [11]. Most interestingly, animal miRNAs target some developmental genes specifically [12] and many miRNAs are expressed in a temporal or tissue-specific pattern [13]. Therefore, miRNAs may be particularly involved in regulating development and function of tissues and organs.

The pig (*Sus scrofa*) is an important animal not only for meat production but also as a model organism for comparative studies [14]. Despite the significant role of the animal, the current miRBase release 17.0 [15] listed only 228 distinct miRNA sequences in pigs [16], [17], [18], which is substantially less compared with human and mouse miRNAs (1424 and 720 respectively). MiRNAs were first studied in pigs by Wernersson et al [19], and Sawera et al. identified the first porcine miRNA cluster [20]. At the beginning, new porcine miRNAs were found using a homology search [21]. With the emergence of microarray and Solexa sequencing, the abundance of porcine miRNA was found, most of them focused on porcine miRNAs from the skeletal muscle and adipose tissuein all life stages [18], [22], [23], [24]. One study focused on the developing brain [17], but none has focused on miRNAs from the pituitary.

The pituitary is a pea-sized gland located at the base of the brain and attached to the hypothalamus by nerve fibers. It is the “master” endocrine organ as it receives signals from the brain and uses these messages to produce hormones that affect many body processes, including animal growth, bone metabolism, and the cell generation cycle [25], [26], [27]. Profiling pituitary miRNAs may thus enable us to elucidate not only how miRNAs are involved in regulating the development and function of the organ but also how miRNAs are involved in regulating the development of the individual or species characteristics of an animal. Thus far, only a few studies have addressed the involvement of miRNAs in the functions of the pituitary; miR-26 has recently been reported to be critical for anterior pituitary development [28], and most others are related to the development of tumors. For example, miR-15 and miR-16 were found to be down-regulated in pituitary adenomas and correlated with the secretion of P43, a precursor of the inflammatory cytokine endothelial monocyte-activating polypeptide II [29]. The expression of the miR-30 family strongly increased while miR-26a and miR-212 were strongly down-regulated in the pituitary gland with ACTH-secreting adenomas [30]. miRNAs actions in the pituitary remain largely unknown.

We conducted an investigation of miRNAs in the porcine pituitary through microarray, Solexa sequencing and real-time PCR. With these powerful technologies, we have identified a large number of porcine miRNAs that are not registered in the *Sus scrofa* miRBase and some new potential porcine miRNAs that have not yet been identified in any species. We have obtained for the first time a comprehensive profile of miRNAs in the porcine pituitary, which provides fundamental information on the miRNAs actions in this “master” endocrine organ.

## Results

### miRNAs and their expressions in the porcine pituitary detected by microarray assay

We applied miRCURY™ LNA Arrays to examine the expression of miRNAs in the porcine pituitary. The chips contain probes for 2500 miRNAs identified in all species including the pig but excluding humans, rats, mice and viruses. With this microarray, we detected a total of 418 miRNAs in the porcine pituitary. According to the miRBase17.0, we have detected 154 known porcine miRNAs (Table 1) and 264 conserved porcine miRNAs by the microarray assay (Table 2). Among the detected miRNAs, 53 had an expression signal greater than 1, 26 reached a signal greater than 2, and 18 reached a signal greater than 3, each with the miR-7 on the top (16.8), which was significantly higher than the expression in skeletal muscles and adipose tissues (data unpublished). These enriched miRNAs should be the main active players of miRNA regulation in the porcine pituitary.

**Table 1:** Known porcine miRNAs# detected in pituitary by microarray.

miRNA	signal	miRNA	signal	miRNA	signal	miRNA	signal
miR-7	16.7999	miR-500	0.7153	miR-181d	0.1607	miR-499	0.0394
let-7a	9.1408	miR-149	0.6334	miR-98	0.1460	miR-455	0.0378
miR-125b	8.6723	miR-99a	0.5987	miR-365	0.1460	miR-542	0.0368
let-7c	7.8025	miR-143	0.5704	miR-15b	0.1366	miR-532	0.0368
miR-125a	7.2379	miR-432	0.5625	miR-128	0.1350	miR-217	0.0362
let-7e	6.8955	miR-152	0.5242	miR-378	0.1308	miR-652	0.0357
miR-26a	5.3472	miR-23b	0.5158	miR-324	0.1297	miR-92a	0.0347
miR-29a	4.5877	miR-429	0.5116	miR-204	0.1292	miR-155	0.0320
miR-30a	4.1003	miR-184	0.5084	miR-146b	0.1261	miR-122	0.0310
miR-22	3.1544	miR-423	0.4932	miR-708	0.1218	miR-342	0.0268
let-7i	3.1497	miR-101b	0.4932	miR-1306	0.1203	miR-376a	0.0263
miR-30b	2.8398	miR-15a	0.4874	miR-338	0.1171	miR-195	0.0252
miR-135a	2.4706	miR-221	0.4706	miR-133b	0.1145	miR-505	0.0236
miR-491	2.1712	miR-758	0.4690	miR-193a	0.1124	miR-18a	0.0236
let-7g	2.1460	miR-28	0.4585	miR-140	0.1019	miR-34c	0.0215
miR-191	2.0021	miR-362	0.4554	miR-95	0.1003	miR-328	0.0184
miR-30c	1.9685	miR-503	0.4501	miR-424	0.0977	miR-27a	0.0168
miR-136	1.6886	miR-339	0.4443	miR-10b	0.0961	miR-490	0.0163
miR-335	1.5935	miR-299	0.3803	miR-615	0.0956	miR-1307	0.0126
miR-222	1.5011	miR-185	0.3640	miR-363	0.0956	miR-381	0.0110
miR-101a	1.4816	miR-99b	0.3424	miR-484	0.0903	miR-18b	0.0110
miR-30d	1.4359	miR-382	0.3062	miR-20	0.0872	miR-301c	0.0100
miR-127	1.4359	miR-340	0.3041	miR-885	0.0867	miR-107	0.0095
miR-19b	1.4296	miR-411	0.2983	miR-628	0.0840	miR-301b	0.0084
miR-30e	1.4265	miR-130a	0.2946	miR-19a	0.0783	miR-451	0.0079
miR-23a	1.3829	miR-10a	0.2873	miR-17	0.0772	miR-190	0.0079
miR-100	1.3025	miR-369	0.2742	miR-181c	0.0767	miR-219	0.0063
miR-29b	1.1849	miR-374b	0.2731	miR-130b	0.0709	miR-146a	0.0063
miR-24	1.1239	miR-425	0.2642	miR-106a	0.0678	miR-504	0.0058
miR-151	1.1113	miR-133a	0.2631	miR-1	0.0662	miR-1271	0.0053
miR-361	1.0336	miR-664	0.2384	miR-16	0.0625	miR-183	0.0047
miR-376c	1.0152	miR-450a	0.2384	miR-486	0.0614	let-7f	0.0032
miR-320	0.8824	miR-487b	0.2353	miR-214	0.0572	miR-32	0.0026
miR-331	0.8314	miR-92b	0.2048	miR-202	0.0557	miR-301a	0.0026
miR-186	0.8078	miR-145	0.1943	miR-199b	0.0541	miR-574	0.0021
miR-181a	0.7868	miR-142b	0.1901	miR-383	0.0520	miR-124a	0.0011
miR-21	0.7857	miR-376b	0.1770	miR-9	0.0515	miR-1224	0.0011
miR-148b	0.7847	miR-181b	0.1723	miR-421	0.0499		
miR-494	0.7600	miR-148a	0.1612	miR-323	0.0431		

Note: # porcine miRNAs deposited in miRBase release 17.0.

**Table 2:** Conserved porcine miRNAs# newly detected in porcine pituitary by microarray.

miRNA	signal	miRNA	signal	miRNA	signal	miRNA	signal
miR-997	0.0504	miR-642	0.1056	miR-296-5p	0.1465	miR-1494	0.0152
miR-993	0.0005	miR-638	0.0121	miR-296-3p	0.2337	miR-1493	0.0657
miR-989	0.1660	miR-63	0.0515	miR-285	0.0357	miR-142a-5p	0.1922
miR-987	0.0047	miR-625	0.0053	miR-282	0.0930	miR-133d	0.0032
miR-974	0.1481	miR-611	0.0184	miR-269	0.0625	miR-129	2.5861
miR-965	0.1161	miR-605	0.0625	miR-268	0.0011	miR-126	0.6933
miR-964	0.2143	miR-589	0.0021	miR-265	0.0074	miR-1239	0.4569
miR-962	0.0016	miR-587	0.0074	miR-263	0.0793	miR-1235	0.0242
miR-940	0.1050	miR-582	0.0404	miR-256	0.0457	miR-1232	0.1964
miR-939	0.4013	miR-56	0.2279	miR-255	0.1371	miR-1230	0.2532
miR-936	1.0509	miR-557	0.1597	miR-253	0.0546	miR-1225-3p	0.0110
miR-933	3.3461	miR-552	0.0200	miR-24b	0.0888	miR-1175	0.1801
miR-922	0.3104	miR-550	1.6166	miR-247	0.0100	miR-1174	0.0788
miR-901	1.3209	miR-55	0.5021	miR-246	0.0011	miR-1144a	0.0783
miR-90	0.1660	miR-548b	0.0032	miR-245	0.1318	miR-1066	0.1329
miR-889	0.0310	miR-542-5p	0.0221	miR-243	0.0704	miR-105b	0.0368
miR-887	0.4632	miR-525	0.0704	miR-241	0.0551	miR-83	0.0425
miR-877	0.0226	miR-52	0.1518	miR-237	0.0289	miR-767	0.0315
miR-875	0.0762	miR-519a	0.0068	miR-235	0.1045	miR-649	0.0714
miR-863-3p	0.0383	miR-518b	0.1176	miR-231	0.0011	miR-616	0.2416
miR-82	0.1292	miR-518a	0.0667	miR-230	0.3062	miR-523	0.0021
miR-81	0.0935	miR-513c	0.1124	miR-223	0.0599	miR-519d	0.0095
miR-80	0.0116	miR-513b	0.0362	miR-220d	0.0005	miR-38	0.0961
miR-8	0.4632	miR-513b	0.0236	miR-220b	0.0525	miR-34b	0.0720
miR-7c	2.1050	miR-513a	0.1434	miR-220a	0.0688	miR-2a	0.1213
miR-799	0.0152	miR-508	0.0436	miR-205b	0.3030	miR-260	0.0053
miR-795	0.3803	miR-502a	0.0352	miR-199a	0.2353	miR-240	0.1197
miR-789b	0.0184	miR-501	0.0026	miR-199	0.1738	miR-218a	0.1003
miR-786	0.0074	miR-498	0.1786	miR-198	0.0184	miR-1b	0.0478
miR-765	0.0184	miR-489	0.0142	miR-1841	0.0053	miR-1844	0.2495
miR-757	0.0037	miR-466	0.1854	miR-1832	0.3230	miR-166c	0.1071
miR-748	0.2521	miR-412	0.0042	miR-1824	0.1670	miR-1496	0.2883
miR-738	0.0604	miR-41	0.0011	miR-1822	0.0089	miR-1240	0.0042
miR-735	0.0042	miR-399f	1.0551	miR-17-3p	0.0247	miR-1036	0.1203
miR-731	0.0032	miR-399a	0.0373	miR-166a	0.0557	miR-103	0.8214
miR-73	0.0194	miR-395c	1.0425	miR-1545	0.0110	miR-1023a-5p	0.1014
miR-727	0.0037	miR-375	2.2085	miR-153b	0.0011	miR-1022	0.1775
miR-71c	0.0053	miR-374a	0.8971	miR-1506	0.0047	miR-1013	0.0362
miR-675	0.1549	miR-33	0.0016	miR-1497h	0.1597	miR-1012	0.0956
miR-67-3p	0.0121	miR-31b	0.0074	miR-1497f	0.3839	miR-101	1.0000
miR-668	0.0341	miR-29d	0.0593	miR-1497e	0.0011		

Note: # porcine miRNAs not yet deposited in miRBase release 17.0;

### miRNAs identified in the porcine pituitary via Solexa sequencing

**Figure 1:**
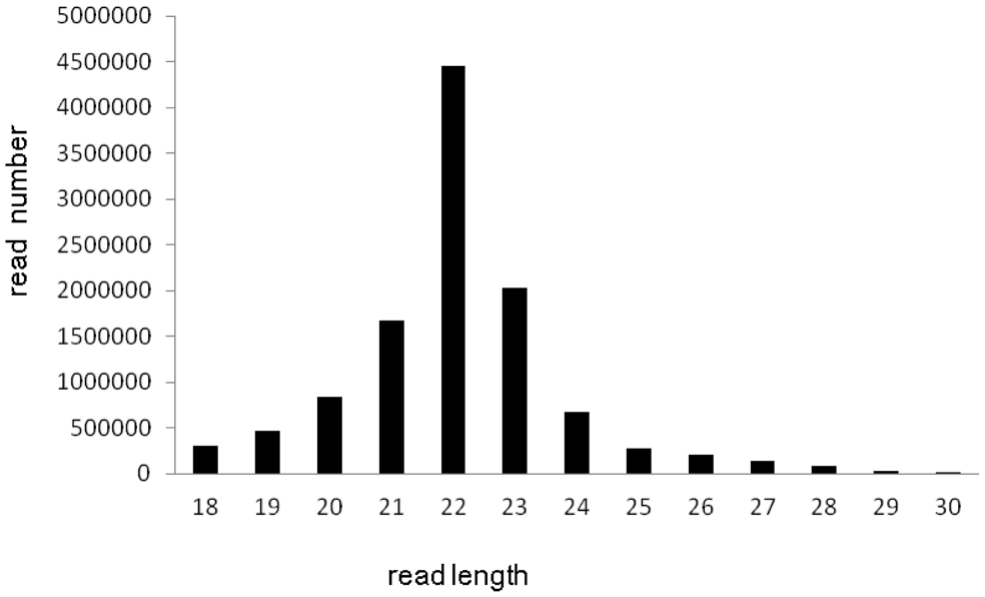
The size distribution of miRNA reads sequenced by solexa. A total of 11,209,341 reads ranging from 18 to 30 base pair [46] in length. The length distribution peaked at 22 bp, which is consisted with commonly expected for miRNAs' length.)

Considering that the miRNAs known in the pig are far fewer than those known in human or mice, and our microarray chips contained only 120 porcine probes, we conducted Solexa sequencing to discover some miRNAs that our microarray might fail to detect and obtain the porcine miRNA sequences that are not yet available. A total of 11,209,341 clean reads were sequenced from the pituitary tissue (Figure S1). These reads contained 744,371 unique sequences, and 109,524 of them can be mapped to the genome (Illumina Genome Analyzer System). After eliminating the unknown and repeat sequences as well as other small RNA reads such as tRNA, rRNA, snoRNA, and piRNA, the remaining 8507 mapped sequences were “blasted” to the miRBase (release17.0) and the matched sequences were annotated according to their similarities with known mature miRNA sequences deposited in the miRBase. As a result, 1312 unique sequences representing 105 mature miRNAs were identified (Table S1), each miRNA include multiple mature variants (Table S2), called isomiRs as other literatures reported [16], [24]. Most of these miRNAs were 18–25 nt long with the peak at 22 nt in the length distribution curve (Fig. 1). Among these cloned and sequenced porcine miRNAs, there were 20 miRNAs that had not been detected by the microarray analysis (Table 3). These miRNAs were either not included in the available commercial microarray chips (underlined in the Table 3), or had a low expression as determined by their low sequencing frequency (lower than 125) except miR-139 (378) and miR-210 (346). Eight miRNAs have not yet been deposited in the *Sus scrofa* miRNA database, but their sequences displayed a perfect or nearly perfect match (mismatch≤1) to orthologous miRNAs of other organisms. They were thus porcine conserved miRNAs. Six of them were identified by Li *et al.* [24], two (miR-2366 and miR-3613) were identified by us for the first time (Table 4). Of them, six have been also obtained from the skeletal muscle and adipose tissues in our previous work (data not published), and the other two miRNAs (underlined in the Table 4) were detected only in the pituitary but not in the skeletal muscle and adipose tissues.

**Table 3:** Conserved porcine miRNAs identified in pituitary by Solexa sequencing but not by microarray analysis.

Name	Count	Name	Count	Name	Count	Name	Count
miR-744	2821	miR-2483	143	miR-450c	28	miR-1296	10
miR-139	978	miR-325	96	miR-215	27	miR-190a*	6
miR-210	346	miR-2366#	274	miR-224	21	miR-326	4
miR-1277	214	miR-206	83	miR-217	13	miR-216	1
miR-1839#	123	miR-760 **#**	55	miR-205	12	miR-3613#	12

Notes: porcine miRNA not yet deposited in miRBase release 17.0;

*miRNA processed from the hairpin arm opposite of the mature miRNA; the underline indicates no probes included in the microarray chips.

**Table 4:** Conserved porcine miRNAs# newly identified in pituitary by Solexa sequencing.

Name	Count	Sequence(5′–3′)	Size	Conservation	Match
miR-31	20578	AGGCAAGATGCTGGCATAGCTGT	23	cfa	perfect
miR-137	2053	TTATTGCTTAAGAATACGCGTA	22	hsa,mmu,rno,dre,fru,mml,oan,prt,bta,eca,tgu,ppy,gga,xtr,mdo,cfa	perfect
miR-660	1961	TACCCATTGCATATCGGAGTTG	22	cfa,mml,hsa,ptr,eca	perfect
miR-1468	317	CTCCGTTTGCCTGTTTTGCTGA	22	bta	perfect
miR-2366	274	TGGGTCACAGAAGAGGGTCTGG	22	bta	perfect
miR-2483	143	AACATCTGGTTGGTTGAGAGA	21	bta	1 nt insersionless
miR-760	55	CGGCTCTGGGTCTGTGGGGAG	20	hsa,mmu,rno,ptr,mml, ppy	perfect
miR-3613-5P	12	TGTTGTACTTTTTTTTTTGTT	21	hsa	perfect

*bta, Bos taurus; cfa, Canis familiaris; dre, Danio rerio; eca,Equus caballus; fru, Fugu rubripes; gga, Gallus gallus;hsa, Homo sapiens; mdo, Monodelphis domestica; mml, Macaca mulatta; mmu, Mus musculus; oan, Ornithorhynchus anatinus; ppy, Pongo pygmaeus; ptr,Pan troglodytes; rno, Rattus norvegicus; tgu,Taeniopygia guttata; xtr, Xenopus tropicalis.*

Note: # Porcine miRNAs not yet deposited in miRBase release 17.0; the underlined miRNAs were detected only in the porcine pituitary but not in the skeletal muscle and in adipose tissues.

The ability of the pre-miRNA sequence to form a canonical stem-loop hairpin structure is one of the critical features that distinguish miRNAs from other small endogenous RNAs [31], [32]. We thus used the remaining 7227 sequences that were mapped to the pig genome but did not match any sequences in miRBase 17.0 to search for potentially new miRNAs by structural reconstruction with their flanking sequence. As a result, 12 potentially new porcine miRNAs were identified by the Mfold and MiReap programs; the precursors can form secondary structures containing stable stem-loops with a minimum free energy less than −20 kcal/mol (Table S3). Among them, ssc-miR-new5 and ssc-miR-new10 has the same seed sequences with miR-2287 and miR-3275, respectively. Other miRNAs represent either porcine-specific or conserved miRNAs not yet discovered in other organisms.

### Validation of miRNA expression via stem-loop real-time PCR

**Figure 2:**
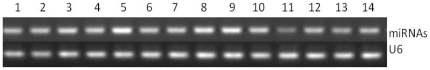
RT-PCR verification of miRNAs detected by microarray and solexa sequencing. The selected miRNAs for the analysis include: miR-99b, miR-204, miR-27a, miR-24, miR-7, miR-145, miR-124, miR-21, miR-125b, miR-30b, miR-128a, miR-122, miR-183, and miR-103.

**Figure 3:**
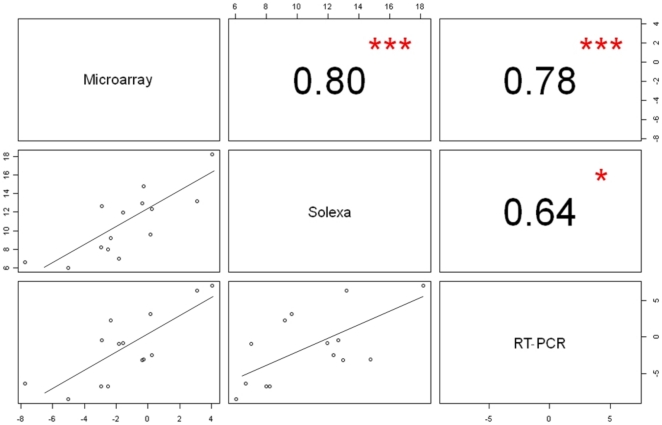
Correlation coefficient matrix for microarray analysis, Solexa sequencing, and RT-PCR. The selected miRNAs for the analysis include: miR-99b, miR-204, miR-27a, miR-24, miR-7, miR-145, miR-124, miR-21, miR-125b, miR-30b, miR-128a, miR-122, miR-183, and miR-103.

To verify and evaluate the reliability of the results from the two platforms, we selected 14 miRNAs for stem-loop real-time PCR assay. These miRNAs were selected from the 81 miRNAs that could be detected by both microarray analysis and Solexa sequencing at different levels of expression. The selection included miR-7, which was ranked at the top for its expression by both methods and miRNAs that had a microarray fold change as low as 0.0047 and a Solexa sequencing frequency count as low as 168. All the miRNAs were successfully detected by the real-time PCR, suggesting that the miRNAs identified by our microarray analysis and Solexa sequencing were reliable for their existence. However, the expression levels detected with the 3 platforms differ to some extent for certain miRNAs. As shown in Figure 2, the expression levels determined by microarray analysis were quite consistent with those determined by real-time PCR assay and Solexa sequencing with Pearson correlation coefficients (R) of 0.80 and 0.78, respectively; whereas the levels determined by Solexa sequencing were a little inconsistent with those determined by real-time PCR (R = 0.64)(Figure 3). This suggested that Solexa sequencing is a more advanced technique for discovering novel miRNAs, but it is somewhat inferior to the microarray method in miRNA quantification.

### Validation of new miRNAs using Northern blot

**Figure 4:**
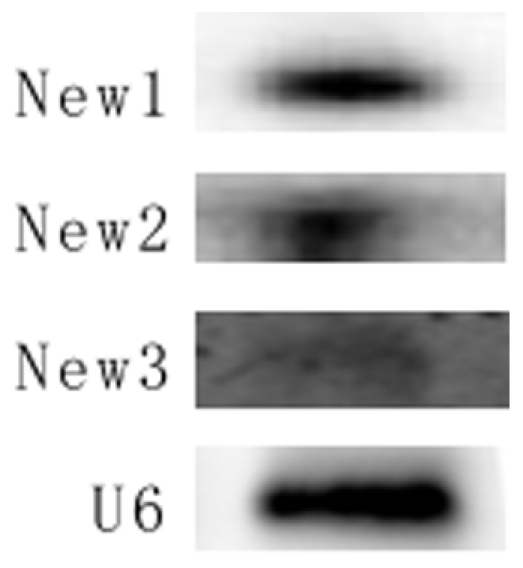
New potential miRNAs in pig pituitary revealed via northern blot. Three new potential miRNAs in pig pituitary were successfully revealed using northern blot, U6 was control. New1, new2 and new3 were listed in Table S3.

The top three highly expressed new miRNAs were confirmed by Northern blot, all of them have been detected in the pituitary tissue (Figure 4).

### miRNA target predictions and KEGG pathway analysis

To gain insight into the general functions of miRNAs in the pituitary, the 10 most abundant miRNAs, except for the let-7 family that is well known to be ubiquitously expressed, were selected for predicting target genes and classified according to KEGG functional annotation using DAVID bioinformatics resources [33]. A total of 2099 target genes were predicted and 12 possible pathways were revealed (Table 5). It appeared that the enriched miRNAs in the pituitary were intensively involved in not only the development of the nervous system such as axonal guidance neurotrophin-signaling, but also the function of the organ such as long-term potentiation and sodium reabsorption. Their roles involved regulations of important inner cell processes such as actin-cytoskeleton regulation, ubiquitin-mediated proteolysis, and regulation of important signaling pathways including MAPK and mTOR pathways and pathways in cancer, as well as regulations of intercellular activities such as focal adhesion.

**Table 5:** Pathways probably regulated by top10 enriched miRNAs in porcine pituitary.

Term	Count	P Value	Benjamini
hsa04360: Axon guidance	42	1.59E-09	2.53E-07
hsa04510: Focal adhesion	53	3.19E-08	2.54E-06
hsa04720: Long-term potentiation	26	1.12E-07	5.96E-06
hsa05200: Pathways in cancer	71	5.67E-07	2.25E-05
hsa04722: Neurotrophin-signaling pathway	35	2.04E-06	6.50E-05
hsa05211: Renal cell carcinoma	23	1.29E-05	3.42E-04
hsa05214: Glioma	21	2.71E-05	6.16E-04
hsa04010: MAPK-signaling pathway	56	2.99E-05	5.95E-04
hsa04960: Aldosterone-regulated sodium reabsorption	16	4.34E-05	7.66E-04
hsa04810: Regulation of actin cytoskeleton	47	5.19E-05	8.25E-04
hsa04120: Ubiquitin-mediated proteolysis	34	5.52E-05	7.98E-04
hsa04150: mTOR-signaling pathway	18	7.15E-05	9.47E-04

Note: Probabilities were evaluated by Bonferroni correction and values less than 1E-03 were considered significant. Count represents targeted genes involved in the term.

## Discussion

In this study, we applied microarray analysis and Solexa sequencing to identify the miRNA expression in the porcine pituitary, and the stem-loop real time RT-PCR to verify and evaluate the data sets obtained via the two high-throughput platforms. We found that the Solexa sequencing could be used to identify novel miRNAs with high accuracy and efficiency. However, microarray analysis appeared to surpass the “next-generation” sequencing methods in quantification. This is also the point of view of Chen et al. and Willenbrock et al. [34], [35]. This limitation of the deep-sequencing approach might be because of the cloning bias or sequencing bias that intrinsically exists in the approach. In addition, multiplexed high-throughput expression sequencing may have affected data quality [35], and fragments with the same seed sequence were regarded as the same miRNA. Further, physical properties or post-transcriptional modifications may make some miRNAs difficult for sequencing. All these factors may decrease the accuracy of the quantification.

By applying two powerful and related technologies, we have obtained a rather comprehensive expression profile of miRNAs in porcine pituitary. The profile included 450 miRNAs, of which 169 were known porcine miRNAs, 269 were conserved miRNAs that can't find in the miRBase porcine data and 12 potentially new miRNAs that have not yet been identified in any species. Of the conserved miRNAs, 106 have been reported in other research [16], [17], [18], [22], [24], and 163 were not yet identified in the pig.

From the known porcine miRNA data, it can be estimated that the porcine pituitary contains at least 74% (169/228) of all miRNA types found in the animal. Although most of them have very low expressions, we have detected 18 miRNAs with more than 3-fold enrichment (signal>3) in the porcine pituitary. Bak *et al.* reported 8 miRNAs (mir-7, mir-7b, mir-375, mir-141 , mir-200a, mir-200c, mir-25and mir-152) with more than 3-fold enrichment in the pituitary of adult mice in a profile of the miRNAs in the central nervous system [36]; and Farh *et al.* and Landgraf *et al.* reported 6 (mir-7, mir-212, mir-26a, mir-191, mir-375 and mir-29) when comparing the miRNAs in normal and tumor pituitary cells [37], [38]. Although those reported data were quite incomplete for miRNA profiles in pituitary; it appeared that miR-7 is the most highly expressed miRNA in the pituitary in all the three species. The up-regulation of miR-141, miR-200a, miR-200c, miR-26a, and miR-29 we detected were also accordant either with mice or with humans. However, there equally were miRNAs that showed discordant in different organisms. For example, miR-375, which was enriched 3-fold in mouse and human pituitary, showed only 2.2-fold in the porcine pituitary; and miR-212 had threefold expression in humans but not in pigs or in mice; miR-25, in mice but not in humans or pigs. These findings indicated that different species could have varying miRNA expressions within the same tissue and/or organ. One should thus be careful in implicating miRNA information from one animal to another. It is certainly one of the most attractive and challenging questions that whether and how such conservation and variation of miRNA expressions are linked to the conserved but varying functions of the pituitaries in different animals or individuals with characterized physiologies.

Compared with skeletal muscles and adipose tissue from the same slaughtered animal (data not published), 19 miRNAs were only detected in the pituitary, suggesting these miRNAs may play important roles in pituitary-specific functions. The most up-regulated miRNA that was expressed in the pituitary relative to skeletal muscle was miR-222, and miR-1 was the most down-regulated. miR-135 was most differently expressed between the pituitary and adipose tissue, as much as 373 folds higher in pituitary, while miR-20a was the most down-regulated one that expressed in the pituitary relative to adipose tissue.

With the Solexa sequencing, four miRNAs, miR-760, miR-1296, miR-137, and miR-362, have been detected only in the pituitary but not muscle or adipose tissue. These four miRNAs also have not been deposited in the *Sus scrofa* database; they were first reported by Li *et al.* using tissues from the whole body [24]. In humans or mice, these miRNAs have been mostly associated with cancer. miR-760 is regulated by the hormone 17-beta-estradiol along with other miRNAs that can target estrogen-responsive transcripts, which is associated with the luminal-like breast tumor [39]. miR-1296 can down-regulate genes of the minichromosome maintenance (MCM) gene family, which are frequently up-regulated in various cancers [40]. miR-137 induces differentiation of adult mouse neural stem cells and stem cells derived from human glioblastoma multiform; it also regulates neuronal maturation by targeting ubiquitin ligase mind bomb-1 protein [41], [42]. The down-regulation of the miR-362 expression may obstruct the development of cultured embryos [43]. Future detail studies are required to reveal the regulation and functions of these pituitary-specific miRNAs. Furthermore, we have identified 12 new potential porcine miRNAs by Solexa sequencing and computational predictions; these miRNAs have not been found in any other species thus far. Further studies of these miRNAs could bring about new insight on miRNA actions.

Finally, the pathway analysis of the top 10 enriched miRNAs has highlighted 12 processes or pathways that the enriched miRNAs may target. These processes or pathways are crucial not only for the development of the nervous system but also for survival, growth, proliferation and motility of the cell, as well as for the development of certain tumors. All these are possible directions for future research on the roles of miRNA regulation in the pituitary.

### Conclusions

We have revealed the existence of 269 porcine conserved miRNAs in addition to those deposited in the current miRBase, of which 154 by array and 2 by Solexa have been newly identified, and we have identified 12 other potentially new porcine miRNAs with their pre-mRNA predicted, which provides important complementary information to the current miRNA database. With the verification and evaluation of the data, we have established for the first time a comprehensive miRNA expression profile of the pituitary gland, which provides fundamental information on miRNA regulation and functions in the porcine pituitary. We found that the pituitary contained particularly many miRNA types relative to its small size and limited cell types in the animal; and miRNAs were dramatically regulated in the pituitary, which showed both accordant and discordant in different species. Actions involving miRNAs in the pituitary are important not only for the development but also for the function of this master endocrine organ, which could contribute to the establishment of individual and species characteristics.

## Methods

### Tissue collection and RNA extraction

Eight 180-day-old Lantang pigs (local strain in China) were slaughtered in a legal slaughterhouse, and the pituitary tissues were collected within half an hour and immediately frozen in liquid nitrogen to ensure RNA quality. Before homogenization, pituitary tissue samples from eight animals were pooled, and total RNA was isolated from the pooled samples by using the Trizol (Invitrogen, USA) reagent according to the manufacturer's protocol. The quality of RNA was examined by 1.5% agarose gel electrophoresis and with BioPhotometer 6131 (Eppendorf, Germany).

### Ethics Statement

All of the animal slaughter experiments were conducted in accordance with the guidelines of Guangdong Province on the Review of Welfare and Ethics of Laboratory Animals approved by the Guangdong Province Administration Office of Laboratory Animals (GPAOLA). All animal procedures were conducted under the protolcol (SCAU-AEC-2010-0416) approved by the Animal Ethics Committee of South China Agricultural University.

### Computational analyses of Solexa sequencing data

All unique reads (744,371 unique in total 11,209,341 sequenced reads were sequenced in our library) were perfect mapped to pig genome using bowtie. The assembed of the pig genome (susScr2, SGSC Sscrofa9.2) were download from UCSC (http://hgdownload.cse.ucsc.edu/goldenPath). Total 7,232,270 (unique 290,998 reads ) can map to genome(5,192,324 reads as 220,778 unique reads mapped to genome uniquely). All reads were align to known miRNA sequences which were download from miRBase (Release16) (http://microrna.sanger.ac.uk/sequences/), ncRNA database which was downloaded from the Rfam database (Release 10.0, http://rfam.janelia.org/), piRNA sequences which were download from RNAdb (http://jsm-research.imb.uq.edu.au/rnadb/default.aspx) respectively. Considering some reads could be mapped to multiple kinds of RNA, the reads can mapped to one kind were removed the dataset which were use to mapped to other sequences.

Map were all allow 0 mismatch, using bowtie with the following Parameter: bowtie -f –v0. Sequences that did not overlap any of these annotations were classified as “unknow”. These “unknow” sequences which mapped at most 5 times to genome were further considered as novel miRNAs candidates. To identify potential miRNA genes, the MIREAP algorithm (http://sourceforge.net/projects/mireap) was employed to obtain all candidate precursors with hairpin-like structures that would perfectly match the sequencing tags [44].

### Microarray assay with miRNA chips

miRCURY™ LNA Array was performed according to the manufacturer's protocol (Exiqon, Denmark). Briefly, after determining the RNA concentration on a NanoDrop instrument, 10 µg of total RNA from the pooled sample was labeled by using the miRCURY™ Hy3™/Hy5™ Power labeling kit and hybridized on the miRCURY™ LNA Array (v.14.0). The array was scanned using the Axon GenePix 4000B microarray scanner. GenePix pro Version 6.0 (Axon Instruments) was used to read the raw intensity of the image. All data used for analysis had a signal-to-noise ratio of >5 and an average sum intensity of 50% higher than that of the background. Expression data were normalized using the lowess (Locally Weighted Scatter plot Smoothing) regression algorithm (MIDAS, TIGR Microarray Data Analysis System), which can produce within-slide normalization to minimize the intensity-dependent differences between the dyes. After normalization, replicated miRNAs were averaged. Differentially expressed miRNAs were identified through Fold Change filtering. Hierarchical clustering was performed using MEV software (v4.6, TIGR).

### Real-time quantification of miRNAs by stem-loop RT-PCR

Six pituitaries were collected from slauthereed Lantang pigs, and total RNA were seperated as discribed above. Stem-loop RT-PCR was performed as previously described [8]. In brief, 1 µg of total RNA from each sample was reverse-transcribed into cDNA by the M-MLV reverse transcriptase (Promega, Guanzhou, China) with looped antisense primers. After 1 hour of incubation at 42°C and 10 min of deactivation at 75°C, the reaction mixes was used as the templates for PCR. Real-time quantitative PCR was performed with standard protocols on a STRATAGENE Mx3005P sequence detection system. The PCR mixture contained 1 µl of cDNA (1∶10 dilution of a RT-reaction mix), 10 µl of 2× SYBR Green PCR Master Mix, 1.5 µM of each primer, and water to make up the final volume to 20 µl. The reaction was performed in a 96-well optical plate at 95°C for 1 min, followed by 35 cycles of 95°C for 15 s, 56°C for 15 s, and 72°C for 40 s. All reactions were run in duplicates, and a negative control without template was included for each gene. The cycle threshold (Ct) was recorded for each reaction and the amount of each miRNA relative to that of U6 RNA was described using the expression 2^−(CtmiRNA−CtU6RNA)^. Primers were designed on the basis of the sequenced miRNA by using Premier 5.0. (Table S4 for primer sequences).

### Northern blot

RNA was extracted from the pituitary tissue of Lantang pig using the same method above. Total RNA was separated on a denaturing 15% polyacrylamideurea gels using TBE buffer, then electroblotted onto the positively charged nylon membrane (Hybond N+ nylon filter, Amersham). After transfer finished, RNA was crosslinked with ultraviolet light (Stratagene). The probes targeting ssc-new-1, ssc-new-2, ssc-new-3 miRNA were labelled with biotin and hybridized to the filter using the MiRNA Northern Blot Assay Kit according to the manufacturer's introduction (Signosis).

### miRNA target predictions and KEGG pathway analysis

miRGen 2.0 database was used to predict the miRNA targets [45]. The predictions were based on human genes as *Sus scrofa* genes are not included in the current versions of miRGen. This approach assumes that the sequences of the miRNA target sites are conserved between orthologues.

The functional annotation of target human genes in the KEGG pathway was performd using DAVID bioinformatics resources [33]. Probabilities were evaluated by Bonferroni correction and values less than 0.001 were considered significant. The relationships between human and pig genes were based on Ensembl release 58 (http://www.ensembl.org/) and retrieved using BioMart (http://www.biomart.org/). We used orthologs between human and pig (S. scrofa) to select the pig miRNA target genes.

### Statistics

Pearson's correlation coefficient R was used to measure the product-moment coefficient of correlation between the quantitative variables. All data were normalized by the Log_2_ (data) transformation. A scatter plot for each paired data sets was then used to analyze the linearity. The Pearson's correlation coefficient R may have any value from 0 to 1. All statistical analyses were performed with the R.

## Supporting Information

Figure S1 **Reads distribution of sRNAs sequenced by solexa.**
(TIF)

Table S1 **Known miRNAs identified in porcine pituitary via Solexa sequencing.**
(DOC)

Table S2 **IsomiR of porcine pituitary.**
(XLS)

Table S3 **Potential new miRNAs# discovered in porcine pituitary.**
(DOC)

Table S4 **Primers used in real-time PCR.**
(DOC)
