# Supplementary figures and images for: A Comprehensive Expression Profile of MicroRNAs in Porcine Pituitary
Source: PLoS One. 2011 Sep 28;6(9):e24883. doi: 10.1371/journal.pone.0024883 (PMC3182167; doi:10.1371/journal.pone.0024883)

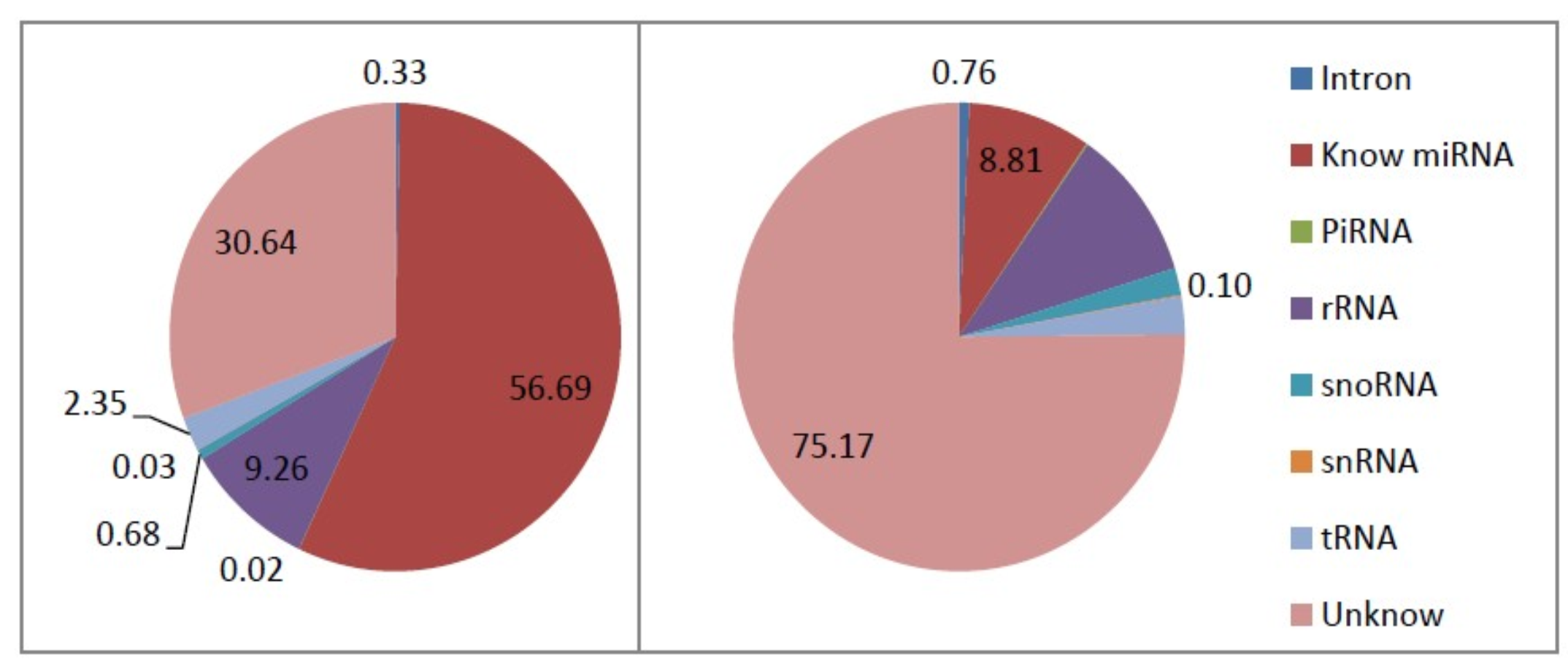

Supplement: Figure S1 — Reads distribution of sRNAs sequenced by solexa. (TIF) [file pone.0024883.s001.tif]
