# Supplementary material for: A Comprehensive Expression Profile of MicroRNAs in Porcine Pituitary
Source: PLoS One. 2011 Sep 28;6(9):e24883. doi: 10.1371/journal.pone.0024883 (PMC3182167; doi:10.1371/journal.pone.0024883)
Supplement: Table S1 — Known miRNAs identified in porcine pituitary via Solexa sequencing. (DOC) [file pone.0024883.s002.doc]

| Name | Count | Name | Count | Name | Count | Name | Count |
| --- | --- | --- | --- | --- | --- | --- | --- |
| miR-7 | 774376 | miR-92a | 10129 | miR-30b | 1396 | miR-146b | 93 |
| miR-101a | 400079 | miR-320 | 8468 | miR-139 | 978 | miR-32 | 85 |
| let-7f | 345979 | miR-15b | 8287 | miR-95 | 869 | miR-206 | 83 |
| miR-30a | 221510 | miR-222 | 5500 | miR-204 | 759 | miR-214 | 59 |
| let-7c | 214420 | miR-186 | 5439 | miR-628 | 728 | miR-20 | 56 |
| miR-99a | 144966 | miR-221 | 4313 | miR-153 | 648 | miR-760 | 55 |
| miR-26a | 92374 | miR-181a | 4308 | miR-181b | 647 | miR-219 | 49 |
| miR-29c | 76123 | miR-323 | 3807 | miR-542 | 536 | miR-184 | 35 |
| miR-103 | 71940 | miR-135 | 3772 | miR-136 | 465 | miR-9-1 | 31 |
| miR-148a | 70224 | miR-486 | 3711 | miR-503 | 414 | miR-9-2 | 31 |
| miR-185 | 44652 | miR-181c | 3472 | miR-210 | 346 | miR-133a | 28 |
| miR-107 | 37362 | miR-1a | 2946 | miR-124a | 335 | miR-450 | 28 |
| miR-140* | 24841 | miR-92b | 2841 | miR-362 | 283 | miR-215 | 27 |
| miR-125b | 24328 | miR-744 | 2821 | miR-491 | 271 | miR-224 | 21 |
| miR-16 | 21890 | miR-130a | 2732 | miR-183 | 260 | miR-18 | 15 |
| miR-21 | 20720 | miR-499 | 2540 | miR-106a | 253 | miR-574-5p | 13 |
| miR-31 | 20578 | miR-296 | 2344 | miR-10 | 232 | miR-217 | 13 |
| let-7i | 16925 | miR-30c | 2073 | miR-1277 | 214 | miR-205 | 12 |
| miR-128 | 16816 | miR-137 | 2053 | miR-1306 | 173 | miR-1296 | 10 |
| miR-23a | 15180 | miR-24 | 2050 | miR-122 | 168 | miR-34a | 10 |
| miR-29b | 13669 | miR-660 | 1961 | miR-19b | 167 | miR-190a* | 6 |
| miR-532 | 12708 | miR-27a | 1901 | miR-17 | 159 | miR-140 | 4 |
| miR-199b* | 12572 | miR-15a | 1651 | miR-1839 | 123 | miR-326 | 4 |
| miR-374 | 10795 | miR-130b | 1623 | miR-500* | 103 | miR-19a | 1 |
| miR-99b | 10361 | miR-145 | 1507 | miR-325 | 96 | miR-216 | 1 |

**Table S1. Known miRNAs identified in porcine pituitary via Solexa sequencing**
