# Supplementary material for: A Comprehensive Expression Profile of MicroRNAs in Porcine Pituitary
Source: PLoS One. 2011 Sep 28;6(9):e24883. doi: 10.1371/journal.pone.0024883 (PMC3182167; doi:10.1371/journal.pone.0024883)
Supplement: Table S3 — Potential new miRNAs# discovered in porcine pituitary. (DOC) [file pone.0024883.s004.doc]

## Table S3. Potential new miRNAs# discovered in porcine pituitary.

| Name | Predicted pre-miRNA* | Count | Location | MEF |
| --- | --- | --- | --- | --- |
| ssc-miR-new1 | 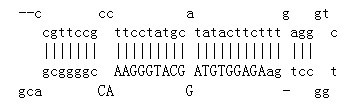 | 257 | Chr14:137881275:137881351:- | -41.4 |
| ssc-miR-new2 | 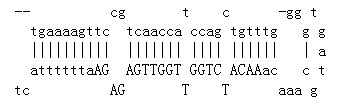 | 68 | Chr1:141259741:141259822:+ | -30 |
| ssc-miR-new3 | 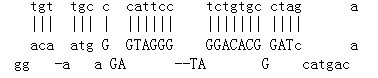 | 38 | Chr13:46697618:46697696:+ | -41.5 |
| ssc-miR-new4 | 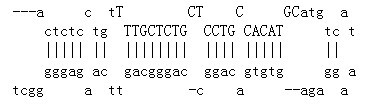 | 26 | Chr7:3324068:3324158:- | -28.2 |
| ssc-miR-new5 | 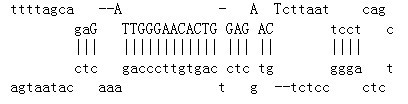 | 21 | Chr1:112779942:112780021:+ | -43.7 |
| ssc-miR-new6 | 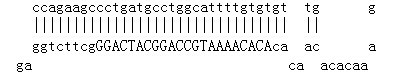 | 18 | Chr7:92498397:92498483:+ | -64.14 |
| ssc-miR-new7 | 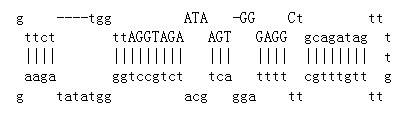 | 11 | Chr7:108303945:108304034:- | -22.1 |
| ssc-miR-new8 | 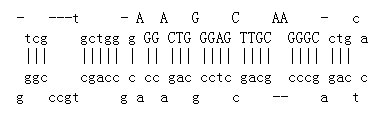 | 10 | Chr7:37425118:37425197:- | -38.6 |
| ssc-miR-new9 | 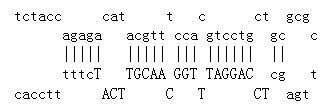 | 9 | Chr5:8273848:8273923:- | -24.9 |
| ssc-miR-new10 | 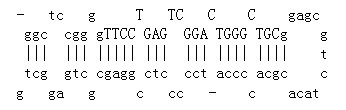 | 8 | Chr4:43267758:43267834:+ | -39.4 |
| ssc-miR-new11 | 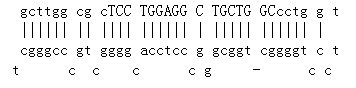 | 7 | Chr11:45806397:45806474:+ | -43.7 |
| ssc-miR-new12 | 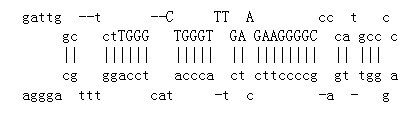 | 5 | Chr7:53953143:53953229:+ | -38.5 |

# Predicted pre-miRNA not yet identified in organisms; *Big letters highlight the sequence obtained by Solexa sequencing
