# Supplementary material for: A Comprehensive Expression Profile of MicroRNAs in Porcine Pituitary
Source: PLoS One. 2011 Sep 28;6(9):e24883. doi: 10.1371/journal.pone.0024883 (PMC3182167; doi:10.1371/journal.pone.0024883)
Supplement: Table S4 — Primers used in real-time PCR. (DOC) [file pone.0024883.s005.doc]

**Table S4. Primers used in real-time PCR**

| Name | RT primers | PCR primers |
| --- | --- | --- |
| ssc-miR-99b | GTCGTATCCAGTGCGTGTCGTGGAGTCGGCAATTGCACTGGATACGACCGCAAGGT | TAATCACCCGTAGAACCG |
| ssc-miR-30b | GTCGTATCCAGTGCGTGTCGTGGAGTCGGCAATTGCACTGGATACGACAGCTGAGA | GCCGCTAAACATCCTACA |
| ssc-miR-27a | GTCGTATCCAGTGCGTGTCGTGGAGTCGGCAATTGCACTGGATACGACGCGGAACT | GAGCTTGTTCACAGTGGC |
| ssc-miR-24 | GTCGTATCCAGTGCGTGTCGTGGAGTCGGCAATTGCACTGGATACGACCTGTTCCT | CGGTTATGGCTCAGTTCAG |
| ssc-miR-21 | GTCGTATCCAGTGCGTGTCGTGGAGTCGGCAATTGCACTGGATACGACTCAACATC | GCACCTAGCTTATCAGAC |
| ssc-miR-204 | GTCGTATCCAGTGCGTGTCGTGGAGTCGGCAATTGCACTGGATACGACAGGCATAG | CGCAGTTCCCTTTGTCATC |
| ssc-miR-183 | GTCGTATCCAGTGCGTGTCGTGGAGTCGGCAATTGCACTGGATACGACCAGTGAAT | GCGTTATGGCACTGGTAGA |
| ssc-miR-145 | GTCGTATCCAGTGCGTGTCGTGGAGTCGGCAATTGCACTGGATACGACAGGGATTC | CGTGTCCAGTTTTCCCAG |
| ssc-miR-128 | GTCGTATCCAGTGCGTGTCGTGGAGTCGGCAATTGCACTGGATACGACAAAAGAGA | CGTCAATCACAGTGAACCG |
| ssc-miR-125b | GTCGTATCCAGTGCGTGTCGTGGAGTCGGCAATTGCACTGGATACGACTCACAAGT | AGACATCCCTGAGACCCTA |
| ssc-miR-124a | GTCGTATCCAGTGCGTGTCGTGGAGTCGGCAATTGCACTGGATACGACTGGCATTC | ACTACTAAGGCACGCGGT |
| ssc-miR-122 | GTCGTATCCAGTGCGTGTCGTGGAGTCGGCAATTGCACTGGATACGACACAAACAC | CGAATGGAGTGTGACAATG |
| ssc-miR-103 | GTCGTATCCAGTGCGTGTCGTGGAGTCGGCAATTGCACTGGATACGACTCATAG | CGCTTGAGCAGCATTGTA |
| ssc-miR-7 | GTCGTATCCAGTGCGTGTCGTGGAGTCGGCAATTGCACTGGATACGACAACAAAT | GCGTGGAAGACTAGTGATTT |
| ssc-miR-29 | GTCGTATCCAGTGCGTGTCGTGGAGTCGGCAATTGCACTGGATACGACACTGATTT | GCCGTAGCACCATTTGA |
| The unique sence primer: TAGTGCGTGTCGTGGAGT | | |
